# Working conditions and Work-Family Conflict in German hospital physicians: psychosocial and organisational predictors and consequences

**DOI:** 10.1186/1471-2458-8-353

**Published:** 2008-10-07

**Authors:** Isabelle Fuß, Matthias Nübling, Hans-Martin Hasselhorn, David Schwappach, Monika A Rieger

**Affiliations:** 1 Department of Occupational and Environmental Medicine, Institute of General Practice and Family Medicine, Faculty of Medicine, University Witten/Herdecke, Alfred-Herrhausen-Str. 50, 58448 Witten, Germany; 2 GEB mbH, Empirical Consulting, Freiburg, Germany; 3 Work physiology, Occupational Health and Infectiology, University of Wuppertal, Germany; 4 Swiss Patient Safety Foundation, Asylstr. 41, 8032 Zürich, Switzerland; 5 Faculty of Medicine, University Witten/Herdecke, Alfred-Herrhausen-Str. 50, 58448 Witten, Germany; 6 Institute of Occupational and Social Medicine, University Hospital Tübingen, 72074 Tübingen, Germany

## Abstract

**Background:**

Germany currently experiences a situation of major physician attrition. The incompatibility between work and family has been discussed as one of the major reasons for the increasing departure of German physicians for non-clinical occupations or abroad. This study investigates predictors for one particular direction of Work-Family Conflict – namely work interfering with family conflict (WIF) – which are located within the psychosocial work environment or work organisation of hospital physicians. Furthermore, effects of WIF on the individual physicians' physical and mental health were examined. Analyses were performed with an emphasis on gender differences. Comparisons with the general German population were made.

**Methods:**

Data were collected by questionnaires as part of a study on *Psychosocial work hazards and strains of German hospital physicians *during April–July 2005. Two hundred and ninety-six hospital physicians (response rate 38.9%) participated in the survey. The Copenhagen Psychosocial Questionnaire (COPSOQ), work interfering with family conflict scale (WIF), and hospital-specific single items on work organisation were used to assess WIF, its predictors, and consequences.

**Results:**

German hospital physicians reported elevated levels of WIF (mean = 74) compared to the general German population (mean = 45, *p *< .01). No significant gender difference was found. Predictors for the WIF were lower age, high quantitative demands at work, elevated number of days at work despite own illness, and consequences of short-notice changes in the duty roster. Good sense of community at work was a protective factor. Compared to the general German population, we observed a significant higher level of quantitative work demands among hospital physicians (mean = 73 vs. mean = 57, *p *< .01). High values of WIF were significantly correlated to higher rates of personal burnout, behavioural and cognitive stress symptoms, and the intention to leave the job. In contrast, low levels of WIF predicted higher job satisfaction, better self-judged general health status, better work ability, and higher satisfaction with life in general. Compared to the German general population, physicians showed significantly higher levels of individual stress and quality of life as well as lower levels for well-being. This has to be judged as an alerting finding regarding the state of physicians' health.

**Conclusion:**

In our study, work interfering with family conflict (WIF) as part of Work-Family Conflict (WFC) was highly prevalent among German hospital physicians. Factors of work organisation as well as factors of interpersonal relations at work were identified as significant predictors for WIF. Some of these predictors are accessible to alteration by improving work organisation in hospitals.

## Background

### Work-Family Conflict (WFC)

Work-Family Conflict (WFC) has been commonly defined as a "form of interrole conflict in which the role pressures from work and family are mutually incompatible in some respect" [1,2]. Its theoretical background is a scarcity hypothesis, which describes that individuals have a certain, limited amount of energy. When involved in multiple roles, these roles tend to drain them and cause stress or interrole conflict [3,4] (in [5]). As conflicts may arise not only between work and family some authors extended the view on other obligations in personal life [6,7].

Individuals identify themselves through social roles. For many people, work and family roles are the most important and self-relevant life roles. As demands and expectations within the family and work domains are not always compatible, conflicts between family and work life can arise. This conflict between work and family is bidirectional in nature; there is work interfering with family (WIF) and family interfering with work (FIW) [8]. WIF occurs when demands and obligations of the work role are deleterious to family life. FIW describes the conflict arising from family obligations that disturb one's work. Family and work as social roles have different grades of permeability: family roles tend to be less structured and more permeable than job roles [9]. This may explain the higher reported frequency of WIF compared to FIW [10,11].

The relationship between work and family life has been investigated over the past years. Different aspects of Work-Family Conflict, its antecedents and consequences as well as gender differences have been examined by various measures and in different samples. As the number of dual-career and single-parent families in Germany has increased over the last decades [12], Work-Family Conflict as a phenomenon in the workforce gains more and more attention.

In the medical sector, Germany currently experiences a situation of major physician attrition which has been attributed to the exit or non-entrance into medical care of young physicians in particular [13]. The introduction of the DRG-based financing system in 2004 has led to major changes in work organisation and condensation of workload for physicians. Additionally in the clinical sector, the number of viable and employed physicians diminished drastically over the last few years [13]. So in 2003, already 21.7% of physicians were without medical occupation [14]. This was mainly due to decrease in medical students and graduates, increased attrition of medical students during their studies and loss of physicians to public health systems of other countries as well as into non-clinical occupations, e.g., health management, pharmaceutical industry, occupational health and safety, research, consulting, or journalism [15,16,14]. In 2004, the German Ministry for Health and Social Security sponsored a study on major reasons for the German physicians' exit from curative medical occupation [14]. 44,4% of the physicians in non-clinical occupations would theoretically like to continue working in hospitals. Major barriers for their return were the following: overwork (52% of male, 54% of female physicians), incompatibility of family and work obligations (37% of male, 54% of female physicians) and insufficient income (51% of male, 24% of female physicians). 82% of physicians working abroad would not like to return to Germany; major reasons for not coming back were good adaptation to the foreign country (70% of male, 72% of female physicians), professional perspectives (69% of male, 65% of female physicians), compatibility between work and family (51% of male, 64% of female physicians) and income (64% of male, 53% of female physicians). Antecedents and sources of Work-Family Conflict among physicians were not investigated in [14].

Already 92,2% of the German medical students expect the conflict between job and family demands to be very high, especially for female physicians [17]. JURKAT ET AL. described the highest expected strains on personal life to be related to childcare and finding time for other activities.

As the ratio of female medical graduates has increased to over 50% in the last 20 years in Germany, Work-Family Conflict could have serious impact on future doctor shortage in Germany leading to a serious threat of the overall quality of medical care [13,14]. Not only in Germany but in most Western countries, the share of female physicians has increased, so that some researchers are talking of a future "pink-collar medicine" [18]. To prevent extreme physician attrition by improving working conditions in particular for women, gender differences of WFC have to be investigated intensively in future.

Physicians hold a profession that is traditionally connected to very high work commitment. Residency, the time of medical specialisation, has been described as the most strenuous and work demanding period during a career as a physician [19]. In this period, physicians mostly experience high work demands with low job control, which may by itself contribute to high job stress [19]. At the same time, residency as a career stage directly follows graduation from medical school, and often coincides with the family-founding life stage of young physicians. Young physicians are more likely to have young children and consequently experience high family or parenting demands, and hence might experience high levels of Work-Family Conflict. On the other hand, female and male physicians who endured the hardship of the time of medical education and residency training [18] may have already given precedence to work over personal activities [20] (in [21]). Data examining the Work-Family Conflict within medical professions is rare in general. Some research has been conducted concerning WFC among nurses [22,23]. Data on WFC among physicians and gender difference in particular is very sparse in general, although DUMELOW observed higher dissatisfaction with work-life balance among female physicians [24]. Physicians' health is underinvestigated in Germany [25]. We are not aware of previous research on details of WFC among German hospital physicians. Our study tries to contribute to a more detailed knowledge of different aspects on WFC.

From the occupational health point of view, in our study we will only examine work interfering with family conflict (WIF) thus focussing on a parameter which may possibly be modified by changes of work-related strains, e.g., work organisation.

• Key Question I a : What is the prevalence of work interfering with family conflict (WIF) among German hospital physicians?

• Key Question I b: Does the prevalence of WIF among German hospital physicians differ from that of the general German population?

• Key Question I c: Which sociodemographic covariates (e.g., age) significantly predict WIF?

According to gender role theory, women are more likely to see their family role as part of their social identity than men do [26,27]. Consequently, WIF is threatening a central social role (and self-identity) in particular for women. Overall Europe, women's roles in the workplace have increased over the last years [12,28], but at the same time expectations within their family roles have not diminished: working mothers still invest more time in family than working men do [29,21,30]. Also in the medical profession this pattern is to be found [31,32] (in [18]). Nevertheless, the situation of women in medicine is somewhat peculiar: for female physicians, it is possible to combine a medical career with child rearing, via the availability of part-time working and the ability to afford high-quality child care [33,34] (in [18]). It still remains unclear if female physicians would report higher levels of WIF. With our study, we want to fill this gap in literature.

Findings of previous research concerning gender difference in WIF and FIW are inconsistent: some studies report higher WIF among women compared to men [29,10,35]. On the other hand, WIF and FIW were found to be similar for men and women by several researchers [26,9,36,3,11,27].

Our study addresses the issue of gender difference in order to bring greater clarity to WIF among German hospital physicians. Details on the prevalence, sources, and outcomes of WIF for female and male physicians carry important implications and high potential for future interventions.

• Key Question I d: Is there a gender difference in the prevalence of WIF among German hospital physicians?

#### Sources of WFC

Various causes for WFC were identified in previous research. These can be assigned to three main sources: the general demands of a role, time devoted to a role, and strains arising from a role [2].

Time-based conflicts occur when "time devoted to requirements of one role make it difficult to fulfill requirements of another" [2]. Time-based sources of WFC within the work domain are for example number of hours worked [29,5,10,11,27], amount and frequency of overtime, work schedule inflexibility [5,35], and presence or irregularity of shiftwork. Time-based family obligations might be marriage or cohabitation with a partner, responsibility for young children, child care hours [29], and spouse employment [2]. FRONE identified *time commitment *as a proximal, direct predictor for WFC. More explicitly, the author states that work time commitment is positively related to WIF, whereas family time commitment is positively related to FIW [8].

Strain-based conflicts occur when "strain from one role makes it difficult to fulfill requirements of another" [2]. Strains arising from the work field are, among others, role conflict, role ambiguity [5], low task autonomy and variety [5], low leader support [2,29], low work group support [29], lack of supportive organisational culture [29], job pressures [29], and physical and mental work demands. Strain-based sources of WFC within the family domain can be low spouse support [5], parental commitment and demands [5], and family conflict [27] such as husband-wife disagreement about family roles. Among others, FRONE described *role-related dissatisfaction or distress *as one of the proximal predictors for WFC: work-related emotional distress leads to increased WIF, whereas family-related emotional distress leads to increased FIW [8]. This has been confirmed by several researchers [3,37,38]. Furthermore, he identified *role overload *as a direct predictor for WFC; this predictor acts via psychological preoccupation, time commitment, emotional distress, and/or physical and psychological fatigue. In detail, work overload is positively related to WIF, whereas family overload is positively related to FIW [8]. More generally, FRONE identified that job stress increased work-to-family conflict, whereas family stress increased family-to-work conflict [9].

One of the most recognised theoretical models concerning stress factors and consequences of perceived stress in work sciences is the "demand-control-support model" [39,40] (in [6]). This model assumes working situations to have negative psychological or physical consequences in particular when high demands coincide with limited decision latitude and low social support at the workplace. According to this model, physicians working in hospitals are likely to perceive high work stress [41]. As described above, the findings by FRONE[ 8,9] support this assumption. In our study, we investigated different aspects of work demands, control, and support, focussing on hospital-specific working conditions.

To our knowledge, research on physicians' psychosocial and organisational work environment and the consequences on the employees' well-being is rare in Germany. In our study, we want to focus on sources and predictors for WIF, predominantly found within the psychosocial and organisational work environment. The different working conditions inquired in our study touch general work demands as well as time- and strain-based conditions. Most of the variables are captured by one standardised instrument, the German version of the Copenhagen Psychosocial Questionnaire (COPSOQ), whereas hospital-specific issues are measured by specific items.

• Key Question II a: What are the psychosocial and organisational sources for WIF within the work domain of physicians?

• Key Question II b: Do male and female physicians differ in these predictors for WIF?

• Key Question II c: Do German hospital physicians differ from the general German population regarding psychosocial and organisational working conditions?

#### Consequences of WIF

If the job role is a threat to the family role, one of a subject's self-relevant roles is endangered. This is the case when work is interfering with family (WIF). It is likely that attitudes towards work are altered due to its impact on family role and therefore a negative appraisal towards the threat occurs [26,42]. In contrast, FIW is more likely to alter attitudes towards the family [26].

Much research has focused on relationships between Work-Family Conflict and several job outcomes. WFC was negatively related to job satisfaction [26,38,42-44], job performance [45,5], and positively related to job stress [22,46] and the intention to leave the job [45,47,42,44,5-46].

WIF can be a source of distress which may have physical and mental consequences, such as higher emotional exhaustion [45]. Abusive alcohol consumption has also been described as an indirect consequence of WFC [48].

Relationships between WFC and indicators of general well-being and quality of life have also been examined. ARYEE described moderate prediction of life satisfaction and marital satisfaction by WFC [5]. Several researchers described the negative relationship between WFC and life satisfaction [38,42,44]. Gender differences in the relationship between WFC and the above described outcomes have been observed. For example, GRANDEY stated that WIF was predictive for women's job satisfaction, but not for men's [26]. In addition, the author suggested that it is likely that women respond with stronger negative attitudes towards their job than men do [26]. YAVAS reports a stronger relationship between WIF and job performance for men than women and a stronger impact of WIF on turnover intentions for women [45]. While research on physicians' health and well-being has increased during the last years, investigations of relationships between Work-Family Conflict and various outcome measures among German physicians do not exist to our knowledge. Thus we address these parameters with regard to WIF in physicians:

• Key Question III a: Which relationships exist between WIF and various job-related outcomes (e.g., job satisfaction, intention to leave, work ability), personal distress (e.g., cognitive and behavioural stress symptoms, personal burnout, general health status), and perceived quality of life (i.e., satisfaction with life)?

• Key Question III b: Are there any gender differences for these outcomes among hospital physicians?

• Key Question III c: Do outcome levels of WIF in hospital physicians differ from those of the general German population?

## Methods

### Research design

The survey was designed as an explorative study, no primary and secondary outcomes were determined beforehand. Significant differences between different groups of physicians, as shown by statistical analysis, helped to generate hypothesis. The study was conducted as a cross-sectional survey using a standardised questionnaire to assess sociodemographic data and the psychosocial work environment, including work organisation and general and mental health of hospital physicians in Germany.

The final questionnaire was distributed together with an informative letter of invitation and a postage-paid return envelope. The cover letter explained that participation in the study was voluntary and anonymous. We did not ask for written consent of the participants in particular, but judged their voluntary participation after having read the informative letter as "informed consent". The respondents were not compensated for participation. Departments with a response rate under 35% were personally contacted by the first author during a department team meeting. The study protocol was approved by the Ethical Committee of the University Witten/Herdecke (no. 15/2005). The ethical aspects were in full agreement with the Helsinki declaration.

### Instruments

In order to secure a hospital-specific design of the survey, unstructured interviews were conducted with experienced hospital physicians at different stages of career (resident, attending, head of department, academic medicine) as well as related medical professions (nurse, nurse scientist). The aim of these interviews was to obtain insight into physicians' work environment in order to construct survey questions regarding hospital-specific aspects. During the development of the standardised questionnaire, two pre-tests were performed. The first pre-test was conducted to eliminate possible inconsistencies or unclear wording of the survey questions (participants: non-medical, university degree (*n *= 11)). The items on hospital-specific issues were reviewed in a second pre-test (participants: German hospital physicians (*n *= 8)). In case a pre-test participant found a survey item unclear, the item was reworded.

#### Sociodemographic data of respondents

Sociodemographic data included gender, year of birth, cultural background, and cohabitation with a partner and/or children under 15 years of age. Details on the professional background included area of speciality, job position, time working within the profession, and type of job contract.

#### Work-Family Conflict

The definition of work interfering with family (WIF) conflict used in our study is based on the work of NETEMEYER ET AL. [49], but the aspect of *family role *has been extended to other nonwork life roles as suggested by NÜBLING ET AL. [47]: "WIF is an interrole conflict in which the general demands of, time devoted to, and strain created by the job interfere with performing family-related responsibilities or other obligations in one's personal life."

The scale used in this survey was the German version [7] of the original WIF instrument by NETEMEYER[ 49]. NÜBLING adapted it to the German language and expanded the instrument items by supplementary phrases to all nonwork or personal activities. NÜBLING reported a Cronbach's alpha of .92 for the German version [7].

The instrument included five items asking for the influence of work on personal or family life (WIF). The response scale was a 5-point Likert scale (from "strongly agree" to "strongly disagree"). The internal consistency of the scale was good (alpha = .92). The WIF scale-value was calculated as the mean of the five item responses transformed to a range from 0–100.

Participants were asked to rate the following five questions for the identification of WIF:

*1. The demands of my work interfere with my home, personal and family life*.

*2. The amount of time my work takes up makes it difficult to fulfill family responsibilities or personal obligations*.

*3. Things I want to do at home do not get done because of the demands my job puts on me*.

*4. My job produces strain that makes it difficult to fulfill family duties or personal duties*.

*5. Due to work-related duties, I have to make changes to my plans for family or personal activities*.

#### Psychosocial work factors and physical and mental health outcome scales (COPSOQ)

The Copenhagen Psychosocial Questionnaire (COPSOQ) [50] was used to assess job-related and psychosocial stress factors at work.

This instrument follows a multidimensional concept and aims to cover the broad demands of the construct "psychological stress at work". It is based on the "demand-control-support model" as described above [39,40] that tries to explain stress as a consequence of high demands at work and simultaneously low control over one's work as well as low social support. Various aspects of work stress, i.e., demands (e.g., cognitive demands), perceived control (e.g., influence at work), and social support (e.g., role clarity) at the workplace are captured by the COPSOQ. An advantage of this survey instrument is its use of scales instead of single items. Most of the scales have been validated and used by many researchers, too. The German version of the COPSOQ [6,7,51] further includes physical and mental health scales (e.g., general health status, work ability) as well as additional outcome scales (e.g., job satisfaction, life satisfaction, burnout). The COPSOQ version used in this survey compromised 20 scales with 70 items, see Additional file 1.

#### Other factors regarding work organisation

Based on unstructured interviews with experienced German hospital physicians and through evaluation of two piloted pre-tests (see above), we generated new single items addressing the specifics of the work environment and work organisation in German hospitals. These assess sources of time-based and strain-based conflicts between work and family as described in the introduction. Items assessing details of demands, control, and support in the hospital were also added to the questionnaire, see Table 1.

**Table 1:** Hospital-specific single items capturing psychosocial working conditions of physicians *(response scales)*

**vocational training**
During the last 12 months, did you take part in a vocational training? *(no; yes, internal training; yes, external training)*
Who paid your last vocational training? *(my superior/employer, I myself, independent sponsors, e.g. industry, research fonds, etc.)*
Was it made possible for you to change your duty for vocational training in the past? *(no; yes, partly; yes, completely)*
**support at work**

Does your superior acknowledge the efforts and results of your work? *(always, often, sometimes, seldom, never/hardly never, I have no superior/no colleagues)*
Do your colleagues acknowledge the efforts and results of your work? *(always, often, sometimes, seldom, never/hardly never, I have no superior/no colleagues)*
How often do you experience competitive pressure among your colleagues? *(always, often, sometimes, seldom, never/almost never, I have no superior/no colleagues)*
**time devoted to a role**

If you worked overtime, would you report it to your superior? *(no, partly, yes)*
How often was the duty roster changed on short-notice (i.e., within 1 to 3 days) during the last 3 months? *(never, around once/twice a month, around once a week, several times a week)*
If the duty roster was changed on short-notice, how often did you have to work on a day off? *(never, less than once a month, more often than once a month)*
If the duty roster was changed on short-notice, how often did you have to work on a weekend off? *(never, less than once a month, more often than once a month)*
If the duty roster was changed on short-notice, how often were your family/personal preferences ignored? *(never, less than once a month, more often than once a month)*
If the duty roster was changed on short-notice, how often did you have to continue working after your duty? *(never, less than once a month, more often than once a month)*
If the duty roster was changed on short-notice, how often did you have to postpone a planned vacation? *(never, less than once in 6 months, more often than once in 6 months)*
In the case of a changed duty roster on short-notice, how stressful was it for you to work on a day off? *(not at all stressful, a bit stressful, very stressful)*
In the case of a changed duty roster on short-notice, how stressful was it for you to work on a weekend off? *(not at all stressful, a bit stressful, very stressful)*
In the case of a changed duty roster on short-notice, how stressful was it for you when family/personal preferences were ignored? *(not at all stressful, a bit stressful, very stressful)*
In the case of a changed duty roster on short-notice, how stressful was it for you to continue working after your duty? *(not at all stressful, a bit stressful, very stressful)*
In the case of a changed duty roster on short-notice, how stressful was it for you to postpone a planned vacation? *(not at all stressful, a bit stressful, very stressful)*
What do you think are the most likely reasons for short-notice changes of the duty roster? *(responsibility for too many patients, mismanagement in duty roster planning, personnel shortage, colleagues on parental leave, colleagues on vacation, colleagues on sick leave)*
**communication**

How often were team meetings (with colleagues and superiors) used as a platform to discuss criticism and improvement strategies? *(never; less than once a month; more often than once a month)*
**outcomes**

During the last 12 months, how often did you go to work despite own illness? *(× times)*

### Statistical analyses

All categorical items described above related to workload, strain, and outcomes derived from the German COPSOQ version were transformed to a scale ranging from 0 (minimum value, e.g., "do not agree at all") to 100 points (maximum value, e.g., "fully agree"). Item non-response and the category "does not apply" were coded as missing values. Scale scores were computed as the mean of the values of the single aspects if at least half of the single items had valid answers. Thus, as all of the single items, all scales therefore have a theoretical range from 0 to 100. Since the transformation is linear, the relation between all items remains unaffected. This transformation is a standard procedure following the recommendation for the German COPSOQ version [6] and allows comparison of our findings with other studies using the same approach.

Data analysis included descriptive statistics, parametric and non-parametric correlation analyses, explorative and confirmatory factor analyses, simple and multiple regression analyses, and reliability analyses. During validation of the original German COPSOQ version [6] in addition to Cronbach's alpha and intra-class-correlation (ICC) for the total population, G-coefficients in a single-facet design [52] were determined in the analysis of the scale reliability to verify the generalisability of the item information [53]. Pearson's chi^2 ^test, correlation analysis (Pearson's *r*), analysis of variance (ANOVA), and linear bivariate and multiple regression analysis were performed. The comparisons between our sample of hospital physicians (*n *= 296) to all German hospital physicians in 2005 [15] (*n *= 146511) was performed through chi^2 ^tests. Significant differences of our sample to the sample of the general German population [6,7] (*n *= 2561) including another sample of German physicians (*n *= 77) were tested through ANOVA. Gender differences were tested also by ANOVA.

All *p*-values given were two-tailed. A *p*-value of less than .05 was considered significant. Values are given as mean and standard deviation (SD). Data were analysed using SPSS V14.0.

### Sample

A survey on *Psychosocial work hazards and strains of German hospital physicians *was conducted during the period of April to July 2005. The study subjects were physicians currently working in two different university teaching hospitals in the region of Northrhine in Germany. The hospitals were almost equal in size. Hospital A has been privately owned since January 2003 whereas hospital B was a new public holding consisting of three formerly independent public hospitals.

Of the 761 physicians who were asked to partake in the survey, 296 responded to the questionnaire (60.1% men, 38.5% women). The overall response rate was 38.9% (Hospital A 35.6%, Hospital B 41.2%) with highest rates in the departments of pediatrics, radiology, anesthesiology, microbiology/laboratory medicine, and urology. In absolute figures, most participants were recruited from the departments of surgery, internal medicine, anesthesiology, and pediatrics (see Table 2). The mean age in the sample was 38.3 years (SD 8.9) with an average time working as a physician of 11.0 years (SD 8.8) and an average age at job entry of 27.3 years (SD 2.5).

**Table 2:** Sociodemographic characteristics of the sample of German hospital physicians, *n *= 296

	absolute (*n*)	relative (%)
**Hospital**		
A	110	37.2
B	186	62.8
**Age cohort**		

up to 34 years	126	42.6
35 – 44 years	98	33.1
45 – 54 years	44	14.9
55 years and older	21	7.1
**Gender**		

male	178	60.1
female	114	38.5
**Professional status**		

resident	128	43.2
board certified specialist	72	24.3
attending	76	25.7
head of department	16	5.4
**Area of specialisation**		

surgery	57	19.3
internal medicine	53	17.9
anesthesiology	48	16.2
pediatrics	46	15.5
small surgical specialities	31	10.5
patient-distant specialities	36	12.2
neurological-dermatological	20	6.8
others/no answer	5	1.7
**Cultural background**		

Germany	277	93.6
foreign country	18	6.1
**Family status**		

living with a partner	221	74.7
living with a child under 15 yrs	114	38.5
**Work contract**		

part-time	35	11.8
full-time	261	88.2
temporary	139	47.0
permanent	155	52.4

General data about physicians working in the participating hospitals were obtained from the human resources department as well the departments' heads. Yet, the representativeness of responders was difficult to determine because there was no complete demographic data available from the hospitals. Gender distribution (chi^2 ^= .005, DF 1, n.s. *p *> .9) and cultural background (chi^2 ^= .39, DF 1, n.s. *p *> .5) in our sample (*n *= 296) were similar to the distribution among German hospital physicians in 2005 (*n *= 146511) [15]. However, responders in our study were slightly younger (mean = 38.3 yrs, SD 8.9 vs. mean = 40.9 yrs, SD not available). Certain medical disciplines were overrepresented in our sample.

## Results

### Work-Family Conflict

#### Prevalence of WIF

Our sample of 296 hospital physicians reached a mean of 74.2 (SD 24.5) and a median of 75.0 points (range 0–100) on the WIF conflict scale (key question I a). There is no cut-off for discriminating between WIF and no-WIF. With regard to the single items of the scale, each 46,1% (*n *= 137) of the respondents completely agreed with the statements "Things I want to do at home do not get done because of the demands my job puts on me" and "Due to work-related duties, I have to make changes to my plans for family or private activities". Only 5,1% (*n *= 15) disagreed with the statement "My job produces strain that makes it difficult to fulfill family duties or personal duties". The remaining items (*job demands*, *time spent at work*, *changes of personal plans*) were important for the majority of physicians, too.

#### Comparison of WIF to German population

Due to the application of the same instruments (German COPSOQ version, WIF) in a study performed in 2003/04 (*n *= 2561, physicians *n *= 77) [6,7], comparisons of our sample with the general German population for WIF (key question I b), psychosocial stressors at work, and outcomes were possible. The results, performed through ANOVA, are displayed partly in Additional file 2. Regarding WIF, hospital physicians participating in our survey had significantly (*p *< .01) higher scores for the WIF scale than the German population and than the very subgroup of physicians surveyed earlier (mean = 59.0, SD 28.3, *p *< 0.01). This finding may indicate an above-average strain concerning work life balance for the physicians in our sample.

#### Sociodemographic predictors of WIF

Bivariate analysis revealed a correlation of several sociodemographic parameters with the WIF scale (key question I c): *age *(*p *< .01), *full-time or part-time work contract *(*p *< .05) and the *medical speciality *(*p *< .05). No significant effects on WIF conflict had *cohabitation with a partner or a child under 15 years of age*, *job position*, *institution (hospital A or B)*, *temporary or permanent work contract*, *cultural background*, *time working within the profession*, and *age at job entry*. In the multivariate regression analysis performed to identify major predictors of WIF (see below), only the sociodemographic factor *age group *stayed significant (*p *< .01): the younger the participants, the higher was the perceived WIF. *Gender *showed no significant difference for WIF (key question I d).

### COPSOQ data

Besides WIF, psychosocial working conditions, general and mental health outcomes were assessed by the German COPSOQ version, see Additional file 2. Thus possible sources of WIF in the psychosocial work environment and general and mental health consequences could be examined (key questions II a – II c, key questions III a – III c).

### Predictors for Work-Family Conflict: bivariate and multivariate analysis

For the identification of significant predictors for WIF (key question II a), we first performed a bivariate analysis (Pearson's correlation) with all sociodemographic variables, the parameters of the psychosocial work environment (COPSOQ scales), and the hospital-specific items depicting work organisation. Secondly, all variables that reached a significance level of *p *< .2 in bivariate analysis were included as potential predictors in the multivariate linear regression model. A step-wise selection procedure with the commonly used including criteria *p *< .05 and the excluding criteria *p *> .10 was applied. A pairwise exclusion of missing values was executed. The procedure followed the suggestions by HOSMER [54]. The estimated values of the final model were compared to empirical means in the survey.

Bivariate analysis revealed several significant correlations between WIF and some of the COPSOQ scales, see Additional file 3. For example higher levels of WIF correlated with higher *quantitative demands *(*p *< .01) and *role conflict *(*p *< .01). Negative correlations were found between *influence at work *(*p *< .01), *sense of community *(*p *< .01), *influence and possibilities for development *(*p *< .01), *social relations and leadership *(*p *< .05), and *quality of leadership *(*p *< .05) and WIF. The scales *commitment to workplace *and *relational justice *did not show significant correlations to WIF.

The hospital-specific items on work organisation were also considered in the bivariate analysis via ANOVA, see Table 3. Positive correlations between WIF and *frequency, suspected reasons*, and *perceived strains *for changes in duty roster on short-term notice were found. The analysis did not reveal any relationship to the following factors: *report of overtime to the superior*, *participation at internal or external advanced professional training*, *the financing of the professional training*, *appreciation of one's work by colleagues*, and the *frequency of team meetings that are used for discussion, criticism, and suggestions for improvement*.

**Table 3:** Correlation between characteristics of work organisation and WIF: bivariate analysis (hierarchy according to eta; +/- indicating positive/negative correlation)

**characteristics of work organisation**		*F*	*p*	eta	eta^2^
number of days gone to work despite own illness	+	16.296	< .001	.386	.146
planned vacations postponed (frequency)	+	13.074	< .001	.291	.085
staff shortage as presumed reasons for changes in duty roster	+	23.098	< .001	.270	.073
strain due to a lost weekend off	+	10.413	< .001	.264	.070
strain due to changes in duty roster ignoring personal requests	+	9.335	< .001	.252	.063
colleagues on vacation as presumed reasons for changes in duty roster	+	14.230	< .001	.215	.046
exemption from duty during training	+	7.688	.001	.225	.051
personal requests ignored at changes in duty roster (frequency)	+	7.374	.001	.221	.049
strain due to being called on duty out of a day off	+	7.063	.001	.221	.049
strain due to having to continue to work after shift	+	6.144	.002	.205	.042
competitive pressure in department	+	4.072	.003	.232	.054
colleagues on maternity/parental leave as presumed reasons for changes in duty roster	+	8.776	.003	.171	.029
general frequency changes in duty roster on short notice	+	4.754	.003	.218	.047
appreciation of work by superiors	-	3.502	.008	.216	.047
continue working after shift (frequency)	+	3.922	.021	.164	.027

The final multivariate regression model included five factors, which together explained 45.2% of the variance (cumulative *r*^2 ^= .45) (see Table 4). Positive predictors for WIF conflict were the scales *quantitative demands *(*p *< .01), *number of days gone to work despite own illness *(*p *< .01), and the *frequency of postponing planned vacations due to changes on the duty roster *(*p *< .01). Individuals who perceived a high workload, went to work despite own illness often, and had to postpone their planned vacation due to duty roster irregularities had higher WIF than persons who did not. For example, physicians that worked despite own illness more than 5 days in the last 12 months had a WIF mean of 85,3 (SD 18.7) compared to a mean of 58,4 (SD 30,1) of those who never did so. Negative predictors were the *age group *(*p *< .01), and the *sense of community *(*p *< .05).

**Table 4:** Predictors for WIF: multivariate analysis of sociodemographic variables, psychosocial work environment (COPSOQ), and hospital-specific items of work organisation

	cumul. *r*^2^	stand. beta	significance
quantitative demands	.32	.46	*p *< .001
number of days gone to work despite own illness	.39	.22	*p *< .001
age group	.42	-.19	*p *< .001
frequency to postpone vacation due to changes on duty roster	.44	.16	*p *< .01
sense of community	.45	-.10	*p *< .05

For all the predictors, the performed estimations of WIF means were satisfying and confirming the final regression model.

#### Gender difference in sources of WIF

We controlled the predictors of WIF derived from multivariate analysis for gender difference via ANOVA (key question II b). The sociodemographic variable *age group *was significantly different between men and women (*p *< .05, eta = .13, eta square = .02): women were significantly younger than men. *Sense of community *was the only predicting COPSOQ scale which was significantly different for women (mean = 78) and men (mean = 72 ; *p *< .01) For none of the other predictors, a gender difference could be identified, see Table 5.

**Table 5:** Significant gender differences found in predictors and outcome variables for WIF

		mean	*n*	sd	*F*	*p*	eta
Work-Family Conflict (WIF)	female	73.73	114	23.099	.021	.886	.01
	male	74.15	177	25.487			
	total	73.99	291	24.539			
**predictor for WIF**							

sense of community	female	77.67	114	15.942	8.976	.003	.17
	male	72.05	178	15.428			
	total	74.24	292	15.843			
**outcome variables for WIF**							

job satisfaction	female	52.38	114	15.914	4.734	.030	.13
	male	56.92	178	18.286			
	total	55.15	292	17.511			
							
Work Ability Index (WAI)	female	77.99	106	13.388	5.139	.024	.13
	male	81.36	176	11.247			
	total	80.09	282	12.183			
							
Copenhagen Burnout	female	51.05	114	17.712	9.731	.002	.18
Inventory (CBI)	male	44.25	178	18.477			
	total	46.90	292	18.453			

Predictors derived from multivariate analysis, outcome variables derived from bi-variate analysis.

#### Comparison of psychosocial work stressors to German population

The two predictors describing the psychosocial work environment (*quantitative demands*, *sense of community*) were compared to the results in the German sample (*n *= 2561) [6,7] and respectively to the German physicians investigated earlier (*n *= 77) (key question II c). The *quantitative demands at work *were significantly higher in our sample (*p *< .01) than in the general population, see Additional file 2. The predictor *sense of community *was not different to the general German population. No difference was found regarding the COPSOQ scales to the former sample of physicians.

### Outcome factors

We tested the relationship between WIF and job outcomes as well as outcomes measuring physical and mental well-being by bivariate analysis (key question III a).

High values of WIF were significantly correlated with high values of e.g., *intention to leave the job *(*p *< .01), *personal burnout *(*p *< .01) and *behavioural *(*p *< .01) and *cognitive stress symptoms *(*p *< .01), see Table 6. Negative relationships were found between WIF and *life satisfaction *(*p *< .01), *job satisfaction *(*p *< .01), *general health status *(*p *< .01), and *work ability *(*p *< .01).

**Table 6:** Correlation of WIF and outcome variables (scales or indices)

**outcome scales**	Pearson's *r*	*p *(two-sided)	*n *=
behavioural stress symptoms	.58	< .01	294
Copenhagen Burnout Inventory (CBI), personal burnout	.55	< .01	295
satisfaction with life scale (SWLS) (5–35)	-.42	< .01	295
job satisfaction	-.36	< .01	295
intention to leave	.37	< .01	294
cognitive stress symptoms	.31	< .01	295
Work Ability Index (0–100)	-.30	< .01	285
general health status	-.26	< .01	294

#### Gender difference of outcomes

In key question III b we asked for the gender difference of the level of physical and mental well-being and other job outcomes. Our analysis (ANOVA) showed women reporting significantly lower levels of *job satisfaction *(mean = 52.4, SD 15.9, *p *< .05) than men (mean = 56.9, SD 18.3), and rating their *work ability *(mean = 78.0, SD 13.4, *p *< .05) worse than men (mean = 81.4, SD 11.2). Furthermore, on the *personal burnout *scale women scored higher (mean = 51.1, SD 17.7 *p *< .01) than men (mean = 44.3, SD 18.5), see Table 5.

#### Comparison of outcomes to German population

We compared all mental and physical outcomes among the German hospital physicians with the general German population (key question III c), see Additional file 2. Significantly higher values were reached in our sample for the scales *behavioural stress symptoms *(*p *< .01), *personal burnout *(*p *< .01), and the *intention to leave *(*p *< .05). Interestingly, our sample of hospital physicians rated their *general health status *(*p *< .01) and their own *work ability *(*p *< .01) significantly better than the general population. In contrast to that, hospital physicians were significantly less satisfied with their job than the general population (*p *< .01). No difference could be identified for the outcomes *cognitive stress symptoms *and general *satisfaction with life*.

By comparing our sample with the subgroup of physicians surveyed in [6,7], we only found higher scores of the *behavioural stress symptoms *(*p *< .01) than among the physicians investigated earlier.

## Discussion

Over the last few years, Work-Family Conflict has received growing attention as an important phenomenon in work sciences. In addition to its influence on job outcomes such as job satisfaction [55,26,57,41,38-44], job performance [45,5], and intention to leave the job [45,47,42,44,5], several effects on the individual's physical or mental well-being, and quality of life [58-64,45,5,38,42,44] have been reported.

Moreso than for other professionals, the well-being of physicians is important for the health in the wider community in which they work [33]. Stresses and strains of physicians – amongst these the incompatibility between work and family [41,58] – are likely to affect their work performance and thereby the overall quality of medical care [25,33]. Adding to this, Work-Family Conflict is one of the assumed reasons for physicians to exit the curative medical occupation in Germany [14]. Therefore, our study – as a part of research on physicians' health and stress – is of interest to the larger public.

In samples of other professions, findings on gender difference in Work-Family Conflict remain inconsistent: based on gender role theory, some researchers confirm that women are more likely to report higher levels of WFC [29,45,65]. Nevertheless, several studies revealed the contrary [26,9,36,3]. The situation of women in medicine is somewhat peculiar: for female physicians it is possible to combine a medical career with child rearing via the availability of part-time position and the ability to afford high-quality child care [33,34] (in [18]). At the same time, work demands do not appear to be less than for men. Previous findings suggest no gender difference for WFC among physicians [33]. As Work-Family Conflict among hospital physicians has not been a study subject in Germany to our knowledge, it is of major importance to examine this phenomenon in detail.

By using a sample of hospital physicians in Germany (*n *= 296) working in two different institutions, we conducted a cross-sectional study focusing on one direction of Work-family Conflict (WFC): work interfering with family conflict (WIF). The present study aimed to examine psychosocial and organisational predictors for WIF and its effects on various job-related and individual outcomes. Gender differences were examined, too. Furthermore, we compared our findings in physicians with those from the German general population investigated earlier [6]. With our research, we want to contribute to the understanding of physicians' health and working conditions in order to facilitate modifications, e.g., on the level of work organisation.

From an occupational health point of view, we concentrated on the organisational and psychosocial working conditions in our investigation of WIF, as these might be modified more easily than task-inherent work stressors.

### Prevalence of work interfering with family (WIF) conflict

The WIF conflict scale (0–100) showed a high mean among German hospital physicians (mean = 74.2). Our sample of physicians had a significantly (*p *< .01) higher level of WIF compared to the German general population [6,7] (mean = 44.8) as well as to the subgroup of German physicians in that earlier study (*n *= 77, mean = 59.0, SD 28.3, *p *< .01). This finding underlines the relevance of negative work-life balance as an existing stressor for physicians [41,58]. As our study had a cross-sectional design, it has to remain unclear why the present sample showed higher values on the WIF scale than the physicians investigated earlier. It has to be borne in mind that the broad public discussion of physicians' work-life balance in Germany in recent years might have led to a higher awareness with regard to this issue.

In accordance with other research comparing male and female physicians [33], the present study did not find a gender difference for the level of WIF perceived. As a higher WIF level for female physicians was described by DUMELOW [24] and our sample was relatively small, this question should be addressed in future investigations with bigger sample size. In contrast to the results reported in [65,27], we did not observe any effects of cohabitation with a child on WIF which again may be due to the rather small sample size.

### Predictors for WIF

Several sources for WIF conflict could be identified in our study. Consonant with the other research findings [8], our study shows that *quantitative workload *was a significant predictor for WIF. This finding confirms the assumption that work role overload through *quantitative demands *leads to job stress [66,63,23], which may by itself lead to conflict between job and personal life. When comparing the predictors of WIF with data of the German general population [6,7], significantly higher values for the *quantitative demands *in our sample (*p *< .01) were found. In the hospital setting this result is not surprising since condensation of workload in the course of re-structuring of the German health system is one of the most direct effects on the employees, e.g., the physicians.

To date, empirical results suggest that one of the time-based sources of WIF lie in work schedule irregularities [5,23]. We examined frequency, consequences, and strains arising from unpredictable changes of the duty roster among hospital physicians. The frequency of the need to *postpone planned vacations *due to work schedule irregularities was identified as a significantly positive predictor for WIF: the more often a physician had to change vacation plans, the higher was the perceived WIF. Obviously, the demand to give up an important personal "reward" that was planned on long hand creates high conflict between work and personal life. The short-time changes of the work schedule may be rooted in organisational deficits, which could be easily and directly be altered.

The number of days the physicians *went to work despite own health problems or illness *significantly predicted WIF: the more often the respondents ignored their own physical well-being and went to work, the higher was the perceived WIF. This factor has not been described as a source of WIF in prior literature so far. It might reflect a neglect of own physical needs among physicians. Consciously ignoring one's own health might be a national peculiarity, as postponing one's recovery from illness to weekends or holidays seems to be common in the German workforce [67]. On the other hand, the low sickness absence rates of physicians [68] may reflect high work commitment in the medical profession. It has been described elsewhere that the "service ethic" connected to physicians' work demands long hours and the placing of patients' welfare above personal needs [20] (in [21]) [18]. A similar factor, excessive identification with the job, has been described as a predictor for job-related family problems elsewhere, too [8] (in [69]).

In concordance with previous findings [23], *age *was identified as a negative predictor for WIF: physicians under 35 years of age scored much higher on the WIF scale (mean = 79) than physicians aged 55 years and older (mean = 56). A possible explanation could be the general high stress level for young physicians in their first postgraduate years. This may be caused by task-inherent stressors such as dealing with sickness and death, high work demands, little control over assigned duties, and the absence of a work routine. However this could also be attributed simply to the worse working conditions for physicians in lower job positions as described by JURKAT ET AL. [70]. In addition, the time of job entry of young physicians often coincides with the family-founding phase thus leading to conflicting interests in both work- and family-related domains. Furthermore, advanced age could be a protective factor for WIF due to better coping strategies with job stress based on longer job experience [3]. As described by NÜBLING ET AL., different interpretations of our findings could be verified only in longitudinal studies – but remain speculative in cross-sectional surveys [71].

Similar to the results of studies conducted in Germany and the U.S. [72,41,25,29], *sense of community *was identified as the only protective factor for WIF: high sense of community predicted low values of WIF. Our analyses revealed that women rated the sense of community significantly better (mean = 78) than men did (mean = 72).

### Relationship between WIF and outcomes

**Figure 1:**
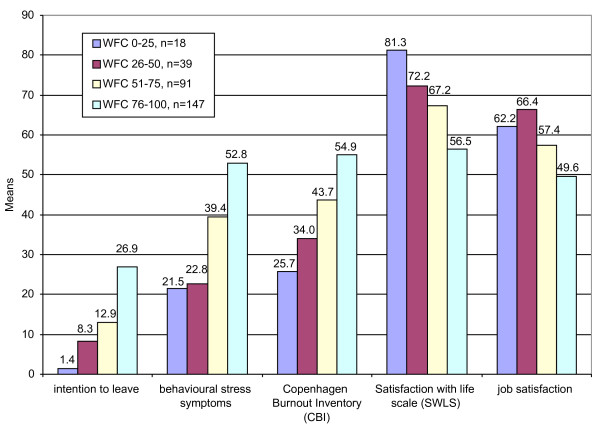
Mean values of outcome variables by WIF level (grouped into quartils).

Our study offers several interesting insights into the possible consequences of WIF conflict: significant relationships to all outcomes investigated were observed. These outcome parameters covered physical and mental health, general well-being, and job-related outcomes. Figure 1 displays the mean values of five outcome parameters according to four quartils covering the WIF scale.

In accordance with the results by HILL [29], who reported a direct relationship between WFC and individual stress, our study showed a positive relationship between WIF and *behavioural and cognitive stress symptoms*. Furthermore, WIF was significantly positively correlated to *personal burnout*. The relationship to emotional exhaustion has been described earlier [45]. In our sample, female physicians had a significantly higher level of burnout than their male counterparts. Previous findings reported the same [73]. According to our results, high WIF was related to high *intention to leave the job *(*p *< .01). This finding is consistent with earlier research, which identified the intention to leave the job as well as high staff turnover as consequences of job-related strains for the family or private life [69,45,47,42,44,5,74]. In a longitudinal study, GRANDEY described chronic WFC to drain resources over time, leading to high intention to leave the job [3]. Furthermore, our result underlines the importance of WIF for the non-entry of – respectively the exit from – the curative medical occupation in German hospital physicians [14].

Similar as in [26,38,42-44,29,23,74], we found a significantly negative relationship to *job satisfaction*: High values of WIF were associated with low job satisfaction. This finding strengthens the assumption that a threat towards a self-relevant role, in this case towards the family or personal life, creates negative attitudes towards the threat, in this case the work [26,42]. To date, empirical results have suggested that women and men have similar levels of job satisfaction [29]. Although SWANSON described a lower job satisfaction for male physicians, in our study female physicians reported a significantly lower job satisfaction than men. In accordance with several researchers [5,38,42,44,29,23], a negative relationship between WIF and *satisfaction with life *could be observed: the higher the WIF, the lower was the satisfaction with life in general.

In our sample, low values of WIF were associated (*p *< .01) with better self-evaluation of the own *health status *and *work ability *(*p *< .01). REIMER ET AL. found that female physicians felt healthier than men [75]. Our findings clearly contradict this view. We found that women felt significantly less able to work than men did.

Almost all results on mental and physical outcomes differed significantly from the general population: physicians in our sample reached significantly higher values for *behavioural stress symptoms *and *personal burnout*. Compared to another sample of physicians [6,7], our sample had a significantly higher mean for *behavioural stress symptoms *(*p *< .01). The higher burnout level was also observed in [63], although HERSCHBACH described only an average burnout-index for physicians [76]. The *intention to leave the job *was significantly higher for our sample than in the general population. This finding might reflect the general climate among physicians, that leaving the medical curative occupation in hospitals is a realistic and attractive opportunity which more and more physicians in Germany consider [15]. Interestingly, despite an identified negative relationship between WIF and *health status *respectively *work ability *(see above) – which would suggest low self-reported health status and work ability in our sample – physicians reported above-average scores on these scales compared to the general population. The mean of *general health status *was 80 in our sample compared to a mean of 72 in the general population. The *work ability *(mean = 80.1 vs. mean = 75.5, *p *< .01) was significantly better in our sample, too. The high scores on these scales could be a result of high social expectations and the existing role model of a "hard-working, but healthy" physician [77]. Other explanations could be over-commitment to the workplace [72], or a very poor perception of own stress and health [77]. Some authors suspect that physicians may have – due to their daily work with very sick and dying people – a distorted relationship to their own body and health, ignoring all signs of own illness [77].

Our findings underline the dissatisfaction of German physicians with their job: our sample had significantly lower *job satisfaction *compared to the general population. Prior research discusses job satisfaction among physicians controversially: results for above-average values [76,57,78] coexist next to those showing below-average values [79] compared to other professions.

A major finding in our study was the above-average value of the work-related stressor *quantitative work demands *and its strong correlation with WIF. The alerting above-average level of all outcome variables points out the current status of physicians' health in Germany. Interestingly, own health concerns, such as going to work despite own illness, significantly predicted WIF, and at the same time physicians reported above-average levels of self-reported health status and work ability.

#### Potential limitations

Potential limitations of the study lie in its cross-sectional design. Exposure to hazardous working conditions and outcomes are measured simultaneously – causal conclusions cannot be drawn. Future research should consider other research designs such as qualitative or mixed method designs, longitudinal studies or non-self-report (e.g., spouse-report) surveys to gather in-depth information on WFC. The overall response rate to our study was 38.9%, which represents an acceptable rate regarding studies in hospital settings. At the same time, this rate carries of course some bias risks. Yet, the sample size might be too small to detect only minor differences between subgroups of physicians or only rather weak correlations between WIF and possible predictors. As sociodemographical data about all employed physicians in both hospitals were incomplete, we neither can make any statements concerning the representativeness of our sample nor non-responders or dropouts. This might implicate the bias that possible very high strains of non-responders were the reason for not participating in the study. Since data were obtained in two hospitals only, general conclusions about hospital physicians in Germany cannot be drawn. As data on psychosocial working conditions were inquired by self-report measures, we cannot claim objectivity of our data. Consequentially, no objective data is available to cross-validate the data of participants.

## Conclusion

The high extent of work interfering with family (WIF) conflict among German hospital physicians revealed in our study underlines the importance of this phenomenon. Our results also identify work organisational and psychosocial factors as predictors for WIF.

Among the factors investigated in our study, one of the major psychosocial hazards experienced by hospital physicians seemed to be the *quantitative workload*. Through better distribution and differentiation of work tasks – time-wise as well as through personnel organisation – this organisational deficit could be handled better in the hospital setting. Measures to be thought of are e.g., the assignment of electronic-supported administrative devices, administrative managers, work flow-charts, division of tasks, interprofessional team-building, management of appointments for in- and out-patients, and better collaboration between the professional groups in the hospital setting [80,77]. In order to suggest more detailed strategies to improve work organisation and working conditions, further investigations are still needed.

*Unpredictable changes of the work schedule *could be revealed as an organisational factor leading to high scores of WIF. The management of duty rosters could easily be altered by better planning and having an alternative plan for unforeseen changes [77]. In this sense, leading personnel should focus on the private needs of the hospital employees – management training could be a further action taken to improve the corporate culture [69]. Good practice examples of the leading personnel itself cannot be underestimated [81]. *Sense of community *was the only factor that seemed to have significant effects with regard to the perception of Work-Family Conflict. Thus, better working atmosphere and teamwork should be encouraged by measures of organisational development in hospitals. Good leadership qualities seem to be indispensable in order to implement a positive atmosphere, improve teamwork, and forward the cutback of hierarchical structures [72].

We observed that especially *young *physicians were more likely to experience WIF conflict than older and more experienced individuals. Previous research suggested the growing workload being the reason for stress and health problems in young employees [81]. Young physicians seem to be susceptible for high stress levels due to their relatively high responsibility and low control at work. At the same time, they are likely to be in the family-founding phase of their lives thus being faced with different demands related to work and private life. To address the needs especially of younger physicians, the start into working life might be improved by e.g., structured vocational training [82], training in communicative and management skills [63], mentoring programs [72], and psychological supervision in order to reduce job-related stress. In addition, the compatibility between career and family must be improved. Flexible work schedules [18,19], job-sharing, and part-time jobs [18,19] are seen to be crucial changes in the work organisation. Clear and consistent parental leave policies [19], together with an open communication about plans for residents' pregnancies should be considered [18,19]. Services such as in-house or supported child care [83,84,19], preparative measures for the reintegration after and continuing education during parental leave are suggested by different authors, too [85,83,86] (in [69]). Especially the provision of child care facilities has been described as an important factor with regard to the correlation of WFC and employment rate of women [83,87]. This aspect should not be underestimated in view of the current physicians' attrition and a high percentage of women medical students in Germany. Satisfaction with child care among German physicians was rated very low in an earlier study [79], especially among female physicians [88].

With regard to reduction of burnout and behavioural and cognitive stress symptoms, continuous education and training on stress-, time-, health- and self-management, and supervision may be helpful for physicians. Workshops on stress-reducing methods [89] and health promoting measures [90] with regular physical check-ups could keep up their motivation. Opportunities for sufficient breaks during work such as fixed or flexible "recreation-schedules" and chill-out rooms should be more and more established [81]. Personal coaching and partnership counselling could help to solve established conflicts between work and family [91]. In some US hospitals, aid-networks for physicians concerning different aspects of family and personal life are implemented, and should be acting as an example for German hospitals [92]. By improving these working conditions, not only WFC could possibly be reduced but also the overall job satisfaction may be increased.

Although interpersonal relations at work seem to have a relevant effect on the perception of Work-Family Conflict among physicians, further scientific efforts should be made to develop organisational concepts for better working conditions for hospital physicians in Germany – for these factors are much more prone to direct alterations.

## Competing interests

The authors declare that they have no competing interests.

## Authors' contributions

IF and MAR developed the conception and design of the study, collected, analysed and interpreted the data, and took primary responsibility for writing the paper. DS, MN, and HMH contributed to the study design. MN provided statistical analysis and advice on how to interpret the results. IF prepared the manuscript and MAR, DS, and MN revised it critically for important intellectual content.

## Pre-publication history

The pre-publication history for this paper can be accessed here:



## Supplementary Material

Additional file 1 **Parameters assessed by the German COPSOQ version**[47,6,7]**in our study, reliability information, sample items, and their response scales.** Click here for file

Additional file 2 **Psychosocial working conditions and outcome factors in the physicians' sample and the general population (2003)**[6,7,47]**assessed by the German COPSOQ version.** Click here for file

Additional file 3 **Full correlation matrix of variables depicting psychosocial work environment and outcomes (German COPSOQ version).** Click here for file
