# Supplementary material for: Working conditions and Work-Family Conflict in German hospital physicians: psychosocial and organisational predictors and consequences
Source: BMC Public Health. 2008 Oct 7;8:353. doi: 10.1186/1471-2458-8-353 (PMC2577658; doi:10.1186/1471-2458-8-353)
Supplement: Additional file 1 — Parameters assessed by the German COPSOQ version[47,6,7]in our study, reliability information, sample items, and their response scales. [file 1471-2458-8-353-S1.pdf]

Table 1: Parameters assessed by the German COPSOQ version [6,7,47] in our study, reliability information, sample items, and their response scales

COPSOQ scale	Cronbach's alpha	sample item *(response scale)*
**demands at work**		
quantitative demands	.73	Is your workload unevenly distributed so it piles up? *(always, often, sometimes, seldom, never/hardly never)*
cognitive demands	.69	Does your work require that you remember a lot of things? *(always, often, sometimes, seldom, never/hardly never)*
emotional demands	.72	Does your work put you in emotionally disturbing situations? *(always, often, sometimes, seldom, never/hardly never)*
demands for hiding emotions	.73	Does your work require that you do not state your opinion? *(always, often, sometimes, seldom, never/hardly never)*
**control or influence/development at work**		
influence at work	.79	Do you have a say in choosing who you work with? *(always, often, sometimes, seldom, never/hardly never)*
possibilities for development	.77	Can you use your skills or expertise in your work? *(to a very large extent, to a large extent, somewhat, to a small extent, to a very small extent)*
workplace commitment	.69	Do you enjoy telling others about your place of work? *(to a very large extent, to a large extent, somewhat, to a small extent, to a very small extent)*
**support or interpersonal relations and leadership**		
role clarity	.84	Do you know exactly which areas are your responsibility? *(to a very large extent, to a large extent, somewhat, to a small extent, to a very small extent)*
role conflict	.73	Are contradictory demands placed on you at work? *(to a very large extent, to a large extent, somewhat, to a small extent, to a very small extent)*
quality of leadership	.85	To what extent would you say that your immediate superior is good at work planning? *(to a very large extent, to a large extent, somewhat, to a small extent, to a very small extent)*
sense of community	.80	Is there a good atmosphere between you and your colleagues? *(always, often, sometimes, seldom, never/hardly never)*
**outcome scales**		
behavioural stress symptoms	.86	Please consider each of the following statements and indicate how well the descriptions fit your situation during the past 4 weeks! I have not been able to stand dealing with other people. *(correct, almost correct, somewhat correct, only slightly correct, incorrect)*
cognitive stress symptoms	.86	How much of the time during the past 4 weeks have you found it difficult to think clearly? *(always, often, sometimes, seldom, never/hardly never)*
relational justice [93–95]	.88	Your supervisor considers your viewpoint. *(I strongly agree , I rather agree, I don't know, I rather disagree, I strongly disagree)*
intention to leave profession [96]	single item	
job satisfaction	.71	Regarding your work in general. How pleased are you with the way your abilities are used? *(very satisfied, satisfied, unsatisfied, highly unsatisfied, not relevant)*
work ability [97,98]	index	How do you rate your current work ability with respect to the physical demands of your work? *(very poor – very good, 5 point Likert scale)*
general health status [99]	single item	
personal burnout [100]	.90	How often do you feel tired? *(always, often, sometimes, seldom, never/hardly never)*
satisfaction with life scale [101]	.87	I'm satisfied with my life. *(I strongly agree – I strongly disagree, 7 point Likert scale)*
