# Supplementary material for: Working conditions and Work-Family Conflict in German hospital physicians: psychosocial and organisational predictors and consequences
Source: BMC Public Health. 2008 Oct 7;8:353. doi: 10.1186/1471-2458-8-353 (PMC2577658; doi:10.1186/1471-2458-8-353)
Supplement: Additional file 2 — Psychosocial working conditions and outcome factors in the physicians' sample and the general population (2003)[6,7,47]assessed by the German COPSOQ version. [file 1471-2458-8-353-S2.pdf]

Table 4: Psychosocial working conditions and outcome factors in the physicians´ sample and the general population (2003) [6, 7, 47] assessed by the German COPSOQ version

	**general German population, COPSOQ 2003/2004**			**hospital physicians 2005**						
**COPSOQ working conditions**	mean	$n =$	sd	mean	$n =$	sd	F	p	eta	eta^2
work-family-conflict	44.8	2541	28.4	74.2	295	24.5	292.203	.000	.306	.093
quantitative demands	56.7	2538	17.2	72.6	296	14.8	233.137	.000	.276	.076
influence at work	46.6	2541	17.9	37.8	295	19.3	62.760	.000	.147	.022
quality of leadership	51.1	2477	23.9	43.5	291	20.6	27.330	.000	.099	.010
role conflict	46.5	2539	19.7	51.0	296	16.3	14.180	.000	.071	.005
commitment to workplace	53.7	2539	19.3	56.6	296	16.1	6.218	.013	.047	.002
role clarity	76.9	2542	15.8	78.5	295	15.1	3.050	.081	.033	.001
relational justice	65.0	2476	23.1	62.7	291	23.8	2.477	.116	.030	.001
possibilities for development	70.3	2541	16.4	71.8	296	15.6	2.268	.132	.028	.001
demands for hiding emotions	52.1	2544	21.2	50.4	296	19.0	1.587	.208	.024	.001
sense of community	75.5	2514	16.9	74.3	296	15.9	1.461	.227	.023	.001
emotional demands	62.6	2537	20.7	64.2	295	16.3	1.456	.228	.023	.001
cognitive demands	74.3	2543	16.0	75.4	296	13.3	1.246	.264	.021	.000
**outcomes**										
behavioural stress symptoms	31.1	2232	22.2	42.9	295	21.1	74.348	.000	.169	.029
general health status	71.8	2427	18.1	80.1	295	14.4	58.176	.000	.145	.021
work ability index	75.5	2363	15.1	80.1	286	12.1	25.017	.000	.097	.009
job satisfaction	59.8	2423	15.7	54.9	296	17.6	24.632	.000	.095	.009
personal burnout	42.2	2434	18.8	46.9	296	18.4	16.974	.000	.079	.006
intention to leave the profession	15.9	1948	22.3	18.7	295	24.5	3.942	.047	.042	.002
satisfaction with life	64.3	2226	19.0	63.5	296	20.2	.405	.525	.013	.000
cognitive stress symptoms	29.4	2230	19.0	29.9	296	17.9	.188	.665	.009	.000
