# Supplementary material for: Working conditions and Work-Family Conflict in German hospital physicians: psychosocial and organisational predictors and consequences
Source: BMC Public Health. 2008 Oct 7;8:353. doi: 10.1186/1471-2458-8-353 (PMC2577658; doi:10.1186/1471-2458-8-353)
Supplement: Additional file 3 — Full correlation matrix of variables depicting psychosocial work environment and outcomes (German COPSOQ version). [file 1471-2458-8-353-S3.pdf]

Table 5: Full correlation matrix of variables depicting psychosocial work environment and outcomes (German COPSOQ version), Ns ranged from 281 to 296. Values on diagonal: Crohnbach's alpha of the scale in our sample, missing values indicate single items of indices. $^{*} = (p < .05)$, $^{**} = (p < .01)$

Variables	1.	2.	3.	4.	5.	6.	7.	8.	9.	10.	11.
1. Work-Family Conflict (WIF)	(.92)										
2. quantitative demands	$.57^{**}$	(.73)									
3. cognitive demands	$.24^{**}$	$.37^{**}$	(.69)								
4. emotional demands	$.28^{**}$	$.30^{**}$	$.38^{**}$	(.72)							
5. demands for hiding emotions	$.15^{*}$	$.17^{**}$	.09	$.15^{*}$	(.73)						
6. influence at work	$-.22^{**}$	$-.02$	$.34^{**}$	$-.00$	$-.31^{**}$	(.79)					
7. possibilities for development	$-.07$	.08	$.45^{**}$	$.25^{**}$	$-.36^{**}$	$.51^{**}$	(.77)				
8. workplace commitment	$-.03$	.10	$.22^{**}$	$.25^{**}$	$-.23^{**}$	$.31^{**}$	$.50^{**}$	(.69)			
9. role clarity	$-.30^{**}$	$-.13^{*}$	$.25^{**}$	$-.07$	$-.27^{**}$	$.42^{**}$	$.44^{**}$	$.20^{**}$	(.84)		
10. role conflict	$.28^{**}$	$.28^{**}$	$.15^{*}$	$.12^{*}$	$.44^{**}$	$-.12^{*}$	$-.18^{**}$	$-.08$	$-.38^{**}$	(.73)	
11. quality of leadership	$-.13^{*}$	$-.20^{**}$	$-.11$	$-.12^{*}$	$-.37^{**}$	$.15^{*}$	$.28^{**}$	$.27^{**}$	$.19^{**}$	$-.33^{**}$	(.85)
12. sense of community	$-.20^{**}$	$-.16^{**}$	$-.11$	$-.05$	$-.29^{**}$	.06	$.20^{**}$	$.16^{**}$	$.26^{**}$	$-.32^{**}$	$.31^{**}$
13. relational justice	$-.11$	$-.14^{*}$	$-.04$	$-.08$	$-.45^{**}$	$.21^{**}$	$.28^{**}$	$.30^{**}$	$.27^{**}$	$.38^{**}$	$.69^{**}$
14. intention to leave the job	$.37^{**}$	$.24^{**}$	.05	$.18^{**}$	$.34^{**}$	$-.25^{**}$	$-.30^{**}$	$-.23^{**}$	$-.28^{**}$	$.23^{**}$	$-.18^{**}$
15. job satisfaction	$-.36^{**}$	$-.21^{**}$	.05	$-.06$	$-.38^{**}$	$.41^{**}$	$.47^{**}$	$.41^{**}$	$.29^{**}$	$-.34^{**}$	$.42^{**}$
16. work ability (0-100)	$-.30^{**}$	$-.22^{**}$	$-.00$	$-.10$	$-.19^{**}$	$.23^{**}$	$.24^{**}$	.10	$.23^{**}$	$-.23^{**}$	$.25^{**}$
17. general health status (0-100)	$-.26^{**}$	$-.20^{**}$	$-.01$	$-.10$	$-.13^{*}$	.08	$.13^{*}$	.00	$.20^{**}$	$-.15^{*}$	$.17^{**}$
18. personal burnout	$.55^{**}$	$.41^{**}$	.07	$.32^{**}$	$.23^{**}$	$-.36^{**}$	$-.16^{**}$	$-.05$	$-.40^{**}$	$.34^{**}$	$-.17^{**}$
19. behavioural stress symptoms	$.58^{**}$	$.41^{**}$	.07	$.26^{**}$	$.25^{**}$	$-.34^{**}$	$-.19^{**}$	$-.09$	$-.43^{**}$	$.36^{**}$	$-.21^{**}$
20. cognitive stress symptoms	$.31^{**}$	$.20^{**}$	$-.10$	.11	$.25^{**}$	$-.27^{**}$	$-.23^{**}$	$-.03$	$-.42^{**}$	$.33^{**}$	$-.12^{*}$
21. life satisfaction	$-.42^{**}$	$-.25^{**}$	.03	$-.16^{**}$	$-.26^{**}$	$.24^{**}$	$.31^{**}$	$.18^{**}$	$.27^{**}$	$-.21^{**}$	$.23^{**}$

continued...	12.	13.	14.	15.	16.	17.	18.	19.	20.	21.
12. sense of community	(.80)									
13. relational justice	$.36^{**}$	(.88)								
14. intention to leave the job	$-.23^{**}$	$-.22^{**}$								
15. job satisfaction	$.28^{**}$	$.39^{**}$	$-.51^{**}$	(.71)						
16. work ability (0-100)	$.22^{**}$	$.29^{**}$	$-.44^{**}$	$.48^{**}$						
17. general health status (0-100)	$.20^{**}$	$.16^{**}$	$-.26^{**}$	$.29^{**}$	$.72^{**}$					
18. personal burnout	$-.23^{**}$	$-.23^{**}$	$.41^{**}$	$-.46^{**}$	$-.58^{**}$	$-.45^{**}$	(.90)			
19. behavioural stress symptoms	$-.28^{**}$	$-.28^{**}$	$.36^{**}$	$-.41^{**}$	$-.49^{**}$	$-.41^{**}$	$.75^{**}$	(.86)		
20. cognitive stress symptoms	$-.16^{**}$	$-.25^{**}$	$.24^{**}$	$-.29^{**}$	$-.43^{**}$	$-.35^{**}$	$.56^{**}$	$.60^{**}$	(.86)	
21. life satisfaction	$.24^{**}$	$.20^{**}$	$-.38^{**}$	$.41^{**}$	$.45^{**}$	$.37^{**}$	$-.48^{**}$	$-.47^{**}$	$-.32^{**}$	(.87)
